# Barriers and Facilitators to Treatment Adherence in Patients with Peripheral Artery Disease: A Qualitative Study of Patients with PAD, Relatives and Healthcare Professionals

**DOI:** 10.2147/PPA.S588692

**Published:** 2026-05-20

**Authors:** Smaragda Lampridou, Layla Bolton Saghdaoui, Gaby Judah, Alun Huw Davies, Mary Wells

**Affiliations:** 1 Vascular Surgery Department, Imperial College Healthcare NHS Trust, London, UK; 2 Faculty of Medicine, Imperial College London, London, UK; 3 ACORN, Guy’s & St Thomas’ NHS Foundation Trust, London, UK

**Keywords:** peripheral artery disease, qualitative research, adherence

## Abstract

**Background:**

Peripheral artery disease (PAD) is a chronic atherosclerotic condition, affecting 20% of adults over 60 in the UK. Guideline-recommended therapy (pharmacotherapy and lifestyle change) has been shown to reduce major cardiovascular and limb events and increase life expectancy; however, adherence is low. Currently, there is a paucity of patient-centred interventions that address all aspects of adherence. Our previous research highlighted that patients’ perceptions of their disease and its treatment strongly influence adherence. However, the views of relatives and healthcare professionals (HCPs) have not been previously explored.

**Purpose:**

To explore the views, experiences, perceived barriers and facilitators to adherence to guideline recommended therapy from the perspectives of patients with PAD, their relatives and HCPs.

**Methods:**

Semi-structured qualitative interviews were conducted with patients with PAD, their relatives and HCPs. Participants were purposively sampled to capture a wide range of perspectives. The Perceptions and Practicalities Approach framed the topic guide and guided analysis. Interviews were recorded, transcribed verbatim, and analysed thematically (inductively and deductively). Stakeholder groups of patients, public and clinicians supported the pilot testing of the topic guide and the data analysis.

**Results:**

Fifteen patients (eight men and seven women, aged 48–82, 8/15 White British), four relatives (all women) and 18 HCPs (five from primary and 13 from secondary care) were interviewed. Seven themes were identified: (1) Knowledge & understanding, (2) Treatment concerns, (3) Motivation, (4) HCP influence, (5) Social & physical context, (6) Everyday reality, (7) Healthcare system factors. Patients, relatives and HCPs did not always align on perceptions.

**Conclusion:**

Our findings illustrate that patients’ adherence to PAD treatment is a complex process shaped by their personal understanding, motivation and daily lives, as well as support from HCPs and the wider system. To enhance adherence, future interventions should focus on improving patient education, addressing misconceptions about the necessity of conservative treatment, fostering individual motivation through practical goal setting, and strengthening collaborative care across the healthcare system.

## Introduction

Peripheral artery disease (PAD) is a chronic atherosclerotic condition affecting over 113 million people worldwide.^1–3^ With ageing populations, both prevalence and burden are expected to rise.^4^,^5^ Guideline-recommended treatment to improve symptoms and delay disease progression combines pharmacotherapy (antiplatelet and lipid-lowering agents) with lifestyle changes focusing on smoking cessation and exercise therapy.^6–8^ Adherence to guideline-recommended therapy reduces major cardiovascular and limb events by 40%, and increases life expectancy by up to six years.^9^,^10^ Yet adherence is often suboptimal,^11^,^12^ increasing the risk of life- and limb-threatening complications.^13^

Previous research on non-adherence mainly focuses on quantitative analyses of sociodemographic and clinical predictors.^14–21^ Factors associated with non-adherence to medications include older age,^18^,^20^ female sex,^18^ employment,^19^ being a new statin user,^19^,^21^ polypharmacy^19^ and the presence of comorbidities.^18^ Experiencing cardiovascular events, such as ischaemic stroke and myocardial infarction, during periods of non-adherence, was associated with a higher probability of subsequent antiplatelet and statin initiation.^14^,^20^ Although prescription initiation by a general practitioner (GP) can improve adherence to antiplatelet medication,^14^ conflicting evidence suggests it may reduce adherence to statins and antihypertensive medications.^16^,^20^ Key barriers to exercise include claudication pain, comorbidities, lack of awareness of exercise recommendations, and limited walking capacity.^22^

In other chronic conditions, patients’ perceptions of illness and treatment strongly influence adherence,^23^,^24^ but there is a paucity of research in PAD exploring patients’ lived experiences and perceptions. Identifying these perceptions can help uncover unexplored barriers and facilitators to adherence. The Perceptions and Practicalities approach (PaPa) distinguishes between perceptual factors (beliefs about disease and treatment) linked to intentional non-adherence and practical (capability and resources) barriers affecting unintentional non-adherence.^25^

The authors of this paper previously conducted two studies to identify adherence determinants in patients with PAD.^12^,^26^ A survey of 105 patients identified an association between negative perceptions about treatment effectiveness and poorer adherence.^12^ A subsequent qualitative study with 12 patients using the PaPa found that concerns about treatment often outweighed perceived benefits, leading to non-adherence.^26^ However, this work involved a small sample and focused only on patient views.

To ascertain the barriers which may affect adherence in patients with PAD, and to develop interventions to overcome them, it is important to understand the perceptions of patients, their relatives and healthcare professionals (HCPs). To date, there are no qualitative studies exploring relatives’ and HCPs’ views and beliefs regarding treatment adherence for PAD. Their experiences, expertise and day-to-day tasks/practice in supporting and managing patients with PAD will be invaluable in identifying and addressing potential barriers.

This study aimed to explore the views, experiences, perceived barriers and facilitators to adherence to guideline-recommended therapy from the perspectives of patients with PAD, their relatives and HCPs. Findings will inform the development of an intervention to improve adherence to the PAD treatment plan.

## Methods

### Design

This was a qualitative study using semi-structured interviews informed by the PaPa Framework. This study adopts an interpretivist paradigm, drawing on hermeneutics and phenomenology to explore how individuals understand their experiences.^27^,^28^ The study received ethical approval from the Health Research Authority (HRA) (REC reference 24/WA/0075). The study complies with the Declaration of Helsinki. Participants completed an informed consent form prior to entering the study, which included publication of anonymised responses/direct quotes. The study is reported in line with the Consolidated Criteria for Reporting Qualitative Research Studies Checklist (COREQ)^29^ (Supplementary File 1).

### Setting, Sampling and Recruitment

Patients and relatives were recruited from vascular outpatient clinics at four large teaching hospitals in England. Clinicians were recruited through the four associated NHS sites, social media and professional organisations (eg. Society of Vascular Nurses), ensuring no industry affiliations. Eligibility criteria are shown in Box 1.

**Box 1:** Eligibility criteria for participation

**Patients**: Adults ≥ 18 years Confirmed diagnosis of PAD, documented on medical records Able to participate in an interview **Relatives**: Adults ≥ 18 years A family member or friend nominated by a patient taking part in the study as someone who is supporting them with their adherence Able to participate in an interview **Healthcare professionals**: Adults ≥ 18 years Registered healthcare professional Experience in caring for patients with PAD for ≥ 6 months, through medication reviews and/or regular vascular diagnostics and/or vascular management

A screening survey was used to achieve a purposive, maximum variation sample. Patients of diverse sociodemographic backgrounds, disease severity and adherence levels; and HCPs with different professional backgrounds and role titles, levels of seniority or experience and working within different settings were selected. Eligible participants received a participant information sheet (PIS) and a brief study overview. Patients willing to participate were asked to share the PIS with their nominated relative/friend. Patients were considered adherent if they reported behaviours broadly consistent with recommended PAD management: i) taking all or nearly all of their medications; ii) not smoking; and iii) completing 30 minutes of daily, unsupervised physical activity (eg. walking). However, adherence was not always clear-cut. Some participants met all of these recommendations, while others adhered to some aspects but not others. For this reason, participants were considered as non-adherent in a particular domain if they reported behaviours such as: i) regularly skipping or halving medications, taking treatment breaks, discontinuing their medications; ii) smoking; and iii) not meeting the guideline-recommended exercise requirements.

### Data Collection

All interviews were conducted by the lead researcher (SL, female), cardiovascular nurse specialist (RGN, MSc, PhD candidate), trained in qualitative methodology, over eight months (May-December 2024). Interviews were either in person in private rooms at the collaborating NHS sites, via phone, or Microsoft Teams, and lasted approximately one hour. The interviews were guided by flexible topic guides (Supplementary File 2), informed by the PaPa,^25^ and our previous work,^12^,^26^ and explored diagnoses, treatment plans, and practical and perceptual adherence facilitators and barriers. Recruitment continued until thematic saturation was reached (when no new themes were identified during iterative data analysis^30^,^31^ after four consecutive interviews; and purposive sampling criteria were met). Participants received a £20 voucher to thank them for their participation.

### Data Analysis

Interviews were audio-recorded and pseudonymised before being transcribed verbatim. The transcripts were not returned to the participants for member checking due to the limited time to complete the study. All transcripts were checked against the recordings for accuracy by the lead researcher (SL). Data analysis followed Braun and Clarke’s thematic analysis approach^31^ (using inductive then deductive analysis) and was managed using NVivo QSR International qualitative analysis software (Version 15) (QSR International Pty Ltd, 2018).

The researcher (SL) reviewed recordings and transcripts, noting initial thoughts. Open coding was applied line-by-line to identify relevant data segments, which were grouped inductively into clusters to form potential themes. These themes captured shared meanings across the dataset and were then visually mapped to explore relationships and distinctions. The themes and subthemes were refined through discussion with three experienced qualitative researchers (LBS & MW, female nurse researchers and GJ, female psychologist) until consensus was reached. The analysis continued iteratively, comparing new codes with existing ones and revisiting themes as new interviews were conducted. This iterative approach ensured thematic saturation and allowed further exploration of emerging insights. After the initial inductive analysis, the PaPa was used to deductively organise findings systematically into perceptions and practicalities influencing adherence.

### Reflexivity

Before obtaining consent, the lead researcher (SL) explained her role to participants, emphasising that she was focused on understanding their perspectives rather than offering clinical advice. She had no prior relationships with the participants, no involvement in their care, and had not previously worked with any of the HCPs involved. Participants were reassured that sharing their experiences would not influence their treatment (patients) or employment (staff). Field notes and reflective journals were maintained to document decisions, enhance the research process, and minimise potential bias.^32^ The study team reviewed these records during data analysis to identify and address any biases.

### Patient and Public Involvement & Stakeholder Engagement

Nine patients with PAD (ages 51–72; four women, five men, three from ethnic minority groups) and two relatives formed the PPI group, influencing the study design, data collection, and analysis. A separate stakeholder group (two vascular nurses and two surgeons) reviewed HCP-related documents and themes. Both groups refined the PIS and topic guides, and provided feedback on the preliminary and final themes to verify if themes accurately reflected their experiences.

## Results

### Participant Characteristics

Thirty nine participants were recruited and 37 participants completed the study: 15 patients, four relatives, and 18 healthcare professionals. Despite all patients being invited to nominate a relative, only five did. One patient and one relative withdrew after being consented, due to a medical emergency. Tables 1 and 2 summarise participant characteristics. Of the 15 patients, eight were men and seven women, aged 48 to 82, and 5/15 were from London, 4/15 from Leicestershire and 6/15 from Yorkshire. Most (8/15) were White British, and one non-English speaker participated with a relative who translated. Only three patients (P3, P8, P11) adhered to all treatment domains, while one (P14) was non-adherent to all. Three (P4, P13, P14) did not take prescribed medications, and two (P1, P15) avoided statins due to side effects. Five took statins inconsistently and declined optimal dosing. Three were current smokers (P4, P14, P15), and two (P5, P13) had recently quit. Most (8/15) met the recommended daily exercise goal.

**Table 1:** Patient Characteristics Based on Purposive Sampling Criteria

ID	Gender	Age	Ethnic Group	Deprivation Index Quintile	Years Diagnosed with PAD	Previous Vascular Intervention	Previous CLTI	Medication Adherence	Smoking Status	Meeting the Exercise Goal
P1	Female	72	White British	3	Newly diagnosed - CLTI	Yes	Yes	Not on statins, adherent with aspirin	Ex smoker	Yes (exercises daily)
P2	Male	74	Black or Black - African	2	16 years	Yes	No	Yes – high but not optimised for statins	Ex smoker	No
P3	Male	67	White British	4	15 years	Yes	No	Yes – high	Ex smoker	Yes (exercises daily)
P4	Male	53	Other – Persian	1	Newly diagnosed - CLTI	Yes	Yes	Not taking medications	Smoker	Yes (exercises daily)
P5	Male	85	White British	3	Newly diagnosed - CLTI	Yes	Yes	Yes - high	Recent ex-smoker	Currently bedbound - amputee
P6	Female	58	Black or Black - African	4	Newly diagnosed - CLTI	Yes	Yes (minor amputation)	Yes – high but not optimised for statins	Never	No
P7	Male	59	Asian - Any other Asian Background	1	9 years	Yes	No	Yes – high but not optimised for statins	Ex smoker	No
P8	Female	66	White British	1	19 years	No	No	Yes - high	Ex smoker	Yes (exercises daily)
P9	Female	61	Black or Black - Caribbean	1	8 years	Yes	No	Yes - high	Ex smoker	No
P10	Female	82	White British	5	5 years	Yes	No	Yes – high but not optimised for statins	Ex smoker	Yes (exercises daily)
P11	Male	70	White British	4	13 years	No	No	Yes - high	Never	Yes (exercises daily)
P12	Female	70	Asian - Indian	1	Newly diagnosed	No	No	Yes – high	Never	No
P13	Male	69	White British	5	Newly diagnosed	No	No	Low – not taking any medications	Ex smoker (recent)	Yes (exercises daily)
P14	Male	48	Asian - Indian	3	Newly diagnosed	No	No	Low – not taking any medications	Current smoker	No
P15	Female	72	White British	1	Newly diagnosed	No	No	High adherence antiplatelet – not taking statins	Current smoker	Yes (exercises daily)

**Abbreviations**: PAD, Peripheral Artery Disease; CLTI, Chronic Limb Threatening Ischaemia.

**Table 2:** Relatives’ Characteristics Based on Purposive Sampling Criteria

ID	Gender	Relative of Patient	Relationship with Patient
R1	Female	P3	Wife
R2	Female	P2	Granddaughter
R3	Female	P10	Daughter
R4	Female	P12	Daughter

**Figure 1:**
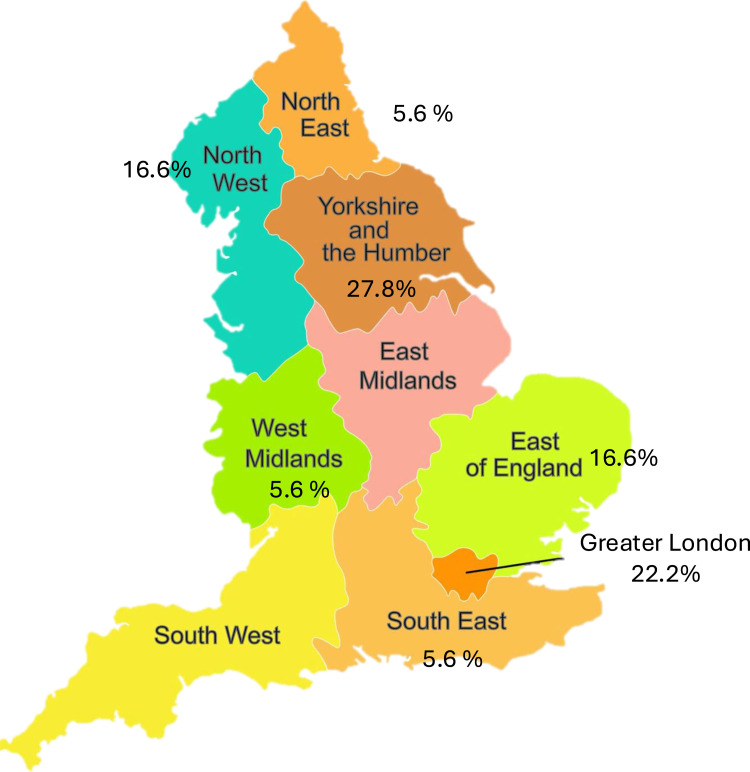
Geographic distribution of HCPs taking part in the study.

Among the 18 HCPs recruited, five worked in primary and 13 in secondary care. The group included four vascular surgeons, three vascular nurses, three podiatrists, three physiotherapists/physiologists, two GPs, two pharmacists, and one vascular scientist. Figure 1 represents the geographic distribution of HCP participants.

### Themes & Sub-Themes Derived from Interviews

**Figure 2:**
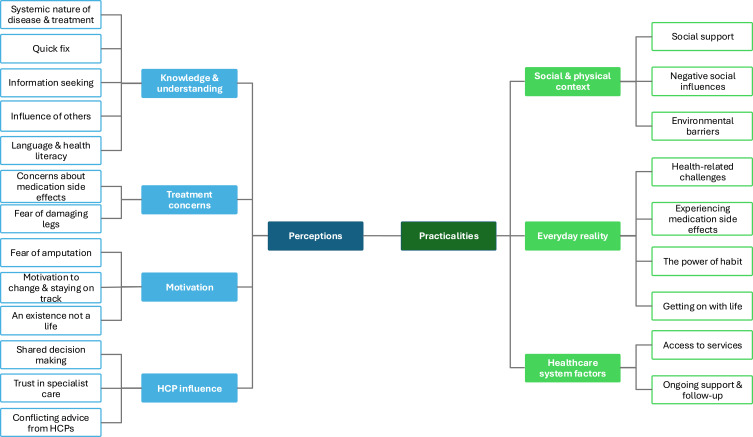
Thematic representation of finding.

Seven themes were identified (see Figure 2), with four mapped to “Perceptions” and three to “Practicalities”. Table 3 presents themes, relevant sub-themes, representative quotes, and whether the findings aligned or differed between different participant groups (patients, relatives and HCPs).

**Table 3:** Themes, Sub-Themes and Relevant Quotes

Themes	Sub-Themes	Quote Examples	Agreement Between Groups
***Perceptions***			
Knowledge & Understanding about disease and treatment	Systematic nature of disease and treatment benefit	“They can’t see that link between their leg and their overall cardiovascular health” (HCP4). “They said that my cholesterol was borderline. But I didn’t understand why I needed to continuously take it” (P6)	Yes
	Quick fix	“I think patients, when they come and you offer them best medical therapy, it feels that it’s perhaps a lesser of the two treatments when you compared to surgical intervention. I do think those patient groups are harder to engage. *I came here with no clinical tablets and now I am on these tablets and now you are gonna try this other medicine and it it’s still, you know, and cannot you just put a stent in? “*(HCP4) “I think it’s an ultrasound which proved that, yes, there’s considerable blockage in both of my legs and he basically said there’s nothing you can do about it. And I was quite shocked as I was sure I was going to have surgery and it will be fixed” (P2)	Yes
	Information Seeking	“I tried to search on the internet. But I found that they gave us too much information. And they are not related. Maybe my condition is not that worse. So for now I will try to walk more and hope for the best” (P4) “Patients usually don’t enough experience at doing Google searches to know the reputable stuff from the stuff that isn’t actually going to be that helpful”. (HCP18).	Yes
	Influence of others	“My neighbour was on aspirin. Next thing I know he’s at the hospital with bleeding and then he died. I’ve seen what happened to him and I don’t want to end up like that”. (P14). “The there’s always the issue with statins in terms of the negative press that’s been around and that tends to be without a typically white British population because obviously they’re the ones reading that information” (HCP3)	No
	Language & Health Literary	“We found the patients from the lowest economic demographics had a poor understanding of their condition, how and when to seek health, take their meds and so on”. (HCP4) “I have to go with her, because she, sometimes, doesn’t understand. Her English isn’t that great, so I go with her”. (R4)	Yes
Perceived treatment concerns	Concerns about medication side effects	“I think the side effects, it will affect me in the long term”. (P4) “I think they all they usually feel quite worried most of the time. I think they’re shocked that they’re going up to such a high dose. And obviously worried about the side effects of that”. (HCP14)	Yes
	Fear of damaging legs	“And the perception of exercise is a big one. The permission to push into pain with claudication is a direct contradiction to the messages around angina in the chest. And yet we would call claudication angina of the leg, in effect, to get some understanding”. (HCP13). “I know the doctors have told me to keep walking with the pain, and I am doing my best, trying to walk everyday, but I sometimes wonder if I am making my leg worse. I am about to have a new stent and I am a bit scared walking after the operation…I just need this stent to work”. (P10) “I’ve been told I need to take them, but I feel like they’re gonna make me feel worse. My leg is already in pain, why should I take something that will cause me more pain? It says in the leaflet, statins can cause leg cramps. I have these already!” (P15)	Yes for exercise, No for statins
HCP influence	Shared decision making	“If patients feel you’re involving them in the decision then they tend to, I think they tend to be more likely to take the medication”. (HCP12) “Don’t ask me, ask the doctors. I don’t know”. (P2)	No
	Trust in specialist care	“Yeah, I think that’s, yeah. And there is something that patients value from hearing it from somebody other than their GP consultant said this and you are like, Oh well that you know that’s true. And that’s what I said but they do value that sort of secondary care opinion”. (HCP18) “I’ll go to the hospital, because there’s no one in my GP that actually knows my problems as much as the hospital does”. (P9)	No
	Conflicting advice from HCPs	“We’re constantly having to have debates about that with the clinicians, which doesn’t help patient understanding when they then get contradicting information”. (HCP15) “Last time I came to the hospital, there’s a doctor there (vascular) who told me I should take double dose, but I do not really listen to the doctor sometimes when they tell me about those tablets. Because I have been taking it for I do not know how many years. My GP said my cholesterol is not high anymore. So, tell me, I should take a double dose now. For what reason?” (P2)	No
Motivation	Fear of amputation	“I think that fear of losing a limb is what means that they will commit to SET”. (HCP6) “I don’t want to lose another thing, so I have to follow my doctors’ advice” (P7, amputee)	No
	Motivation to change and staying on track	“I’ve always been forward-thinking and think before and this helps me stay on track” (P9) “It’s obviously they’ve got to be quite motivated to get the benefits they need to. I think that if a patient is at the right time of their life and they are willing to make changes, they will”. (HCP9)	No
	An existence not a life	“And when I go out, the anxiety and embarrassment, sometimes I start crying with no reason, and I don’t like it in front of people”. (P4) “I think a lot of people with mental health problems like anxiety, depression, once you want to mention a class, they just shut down because they can’t face going into a room with strangers there. They think, no, no, that’s not for me”. (HCP15).	No
***Practicalities***			
Social & physical environment	Social support	“My wife gives me all my medications every morning. Without her, I don’t know how I’d manage”. (P5) “I always think it’s good news if a patient’s got a dog because they then have to go out and walk the dog”. (HCP18)	No
	Negative social influence	“When I used to work, I would go to the pub and have a fag…Everyone was smoking. So even if I wanted to quit, I think it would have been impossible”. (P3) “I think it’s very hard for one person to quit smoking when the other person living in close proximity is still smoking”. (HCP2)	No
	Environmental barriers	“I try to walk everyday, but when it’s raining it’s difficult. It’s slippery, it’s safer to stay inside”. (P11) “I think it’s just being motivated to get out when the weather is terrible, they just can’t be bothered”. (HCP8)	No
Everyday reality	Health-related challenges	“I mean some exercises to do while I’m sitting. Yes, because I also my balance is not great” (P7) “Unfortunately, with our population that we see, there are a lot of other issues and comorbidities. And we see quite a lot of that, that the PAD is only one issue in their lives, and it’s not necessarily the dominant issue until the pain gets really bad. So until then, they do not always focus on PAD” (HCP15).	No
	Experiencing med side effects	“He can’t take certain medications, and it can really mess him up. The aspirin really messed him up, so he can’t take it ever again”. (R2) “In my experience, very few patients actually experience side effects. It’s often more about their perception or reluctance, and we can usually offer alternatives if they genuinely have issues. We have so many statin options, ezetimibe, etc. There are always options”. (HCP4)	Yes
	The power of habit	“I’ve been taking my medications for so long now, it’s part of my life. I never forget to take them” (P11). “Human beings are creatures of habit. I’ve been smoking since I was 12–13 years old. It’s part of my life” (P13, recent ex-smoker)	No
	Getting on with life	“My husband and I used to take the train to London and go shopping, but after his diagnosis he did not feel confident doing that. We started planning our journey differently, allowing more time to get to the station, and taking things slow. He had to accept this new reality and get on with it”. (R1)	No
Healthcare system factors	Access to services	“I suppose it’s just patients being able to access services essentially when they need them”. (HCP10)	No
	Ongoing support and follow-up	“I think I don’t really see what happens to them when they leave clinic. I don’t really know. I don’t really know how well the actual stop smoking sort of interventions in the community work. It’s difficult”. (HCP1) “At the moment I feel that I could do with some one-to-one support in terms of my legs and general fitness level. But I haven’t been offered that”. (P3).	No

### Perceptions

#### Theme 1: Knowledge and Understanding About Disease and Treatment

##### Sub-Theme: Systematic Nature of Disease and Treatment Benefit

Most patients did not recognise PAD as a systemic, chronic condition with cardiovascular implications. Instead, they perceived it as an isolated issue affecting only their limbs. Clinicians highlighted that, based on their experience, patients do not always link PAD with broader cardiovascular risks. This misunderstanding affects adherence to long-term treatment.

And from a patient perspective, the challenges are getting people to understand the severity of their condition in terms of an overall cardiovascular risk. They tend to see it quite separately in terms of the limb and the body as separate symptoms. (HCP2, vascular surgeon, secondary care)

On reflection, I didn’t realise what PAD means and that I could have a heart attack and die. (P10, high antiplatelet adherence, non-optimised for statins, ex-smoker, walks daily)

Patients’ misconceptions about the chronicity of PAD were also identified, with some seeking a definitive cure rather than ongoing management.

They need to find a cure or some sort of injection, or something that will help to regulate the flow of the blood from the arteries. It’s too much taking tablets every day. (P12, high antiplatelet adherence, non-optimised for statins)

This highlights gaps in patient understanding, which can lead to medication non-adherence. Many patients reported stopping medications due to a lack of immediate symptom relief, and even those who adhered daily often questioned the benefit of medications. Overall, most were unaware of the purpose of their medications or how to evaluate their effectiveness.

I was taking the medication, but I wasn’t feeling any better. After a while, I started wondering what the point was, so I just stopped them. (P4, non-adherent to medications)

I don’t really know what my tablets do or how to tell if they’re working. I guess they are doing something, otherwise what’s the point in taking them. (P5, high medication adherence)

Adherent patients appeared to have greater awareness of the medication’s role in maintaining their health. Nonetheless, most had misconceptions about the need for statins, especially if they had normal cholesterol levels. This resulted in skipping or taking lower doses or stopping altogether. All HCPs acknowledged this gap in understanding and they stressed the importance of informing patients of the role of medications and trying to address misconceptions about statins.

They said that my cholesterol was borderline. But I didn’t understand why I needed to continuously take it [statin]. (P6, high antiplatelet adherence, non-optimised for statins and skipping doses)

Despite smoking being a well-established risk factor, smoking cessation was challenging for many patients. Clinicians perceived that patients identified the damage in their legs as irreversible. Hence, they decided to “carry on and take the risk”. For some patients, the reality of disease progression only became evident after severe complications, and even then, it took a major event, such as amputation, before they decided to quit smoking for good.

After they cut my toes, I thought that’s it, I just have to do it [quit smoking]. But it was too late, as then I lost my leg. The doctors told me I had to quit, but I didn’t listen to them. I never thought I would end up like this. (P5, recent ex-smoker)

The role of exercise in PAD management was frequently misunderstood. Some patients reported a lack of awareness of the therapeutic benefits of exercise, particularly those with limited prior engagement in physical activity. Some HCPs also noted that patients with a better understanding of their condition were more likely to participate in SET.

If they haven’t been someone who has exercised a lot in the past, then it can be quite an unusual concept for them that exercise is gonna be treatment. (HCP10, physiotherapist, secondary care)

All patients reported a need for tangible outcomes to help them remain engaged in their treatment. Those who had experienced claudication symptom improvement were more likely to continue exercising. For example: “I carried on walking and it got better and better and better. (P11, exercises daily).

However, some patients became discouraged if exercise did not deliver the expected improvements. Whilst some relatives recognised the value of exercise in improving blood flow, others were not convinced, especially when the patients did not show improvement.

Unfortunately, even the walking didn’t help. So I understand the scenario of yes, you need to walk more to make it better, but unfortunately for [Name] it didn’t. So he then stopped pushing himself to do more and more. (R1, relative of P3)

##### Sub-Theme: Quick Fix

Misunderstanding the chronic nature of PAD often leads to an expectation of a quick fix. Although HCPs regularly discuss the risks and benefits of surgery, many patients and relatives still believe surgical intervention is the cure for PAD. Clinicians noted that patients expect immediate results and often assume that referral to vascular services means they will receive a procedure to restore normal circulation. When surgery is not offered and lifestyle changes or medication are recommended instead, patients may feel “scammed” and disengage with their treatment. Some HCPs also reported that patients are “romanticising the idea about stenting, as it’s not available for everyone”.

I think patients, when they come and you offer them best medical therapy, it feels that it’s perhaps a lesser of the two treatments when compared to surgical intervention. I do think those patient groups are harder to engage. I came here with no tablets and now I’m on these tablets and now you’re gonna try this other medicine and it it’s still, you know, and can’t you just put a stent in? (HCP4, vascular surgeon, secondary care)

Similarly, as many patients and their relatives reported perceiving surgery as the “cure”, they do not believe they require lifelong medications and lifestyle modifications. Some patients and their relatives reported “reading on the internet about getting a stent and going back to normal”, expecting this to be offered to them by their vascular surgeon. Having these expectations and beliefs, some patients reported being frustrated and struggling to see why a surgical intervention was not recommended, as it seemed like a logical solution for them following diagnostic testing.

I think it’s an ultrasound which proved that there’s considerable blockage in both of my legs and he [doctor] basically said there’s nothing you can do about it. I was quite shocked as I was sure I was going to have surgery and it will be fixed. (P2, high antiplatelet adherence, non-optimised for statins, ex-smoker, does not walk daily)

##### Sub-Theme: Information Seeking

Patients reported receiving no information or non-lay information, contributing to non-adherence. Although HCPs said they provided leaflets, most patients did not recalled receiving them, despite some being recruited from the same Trusts. Clinicians stressed the need for clearer, standardised and evidence-based materials. Some patients searched online, mainly using Google or the NHS website, which could hinder or facilitate adherence, depending on the information. Although some patients reported trying to “read a couple of articles before coming to a conclusion”, others did not check whether the information was credible. Some patients reported that social media groups were supportive, with two specifically mentioning that the PAD Facebook group provided positive reinforcement of the benefits of exercise.

I didn’t know that, if you exercised, then it would improve your legs. Nobody said that, so I’ve never done that. I work, so I’ve run upstairs and things like that. I joined the PAD Facebook group and people there said that you must exercise to get better. Now I push myself because I know it’s making me better. (P8, exercises daily)

Patients usually don’t have enough experience at doing Google searches to know the reputable stuff from the stuff that isn’t actually going to be that helpful. (HCP18, GP, primary care)

##### Sub-Theme: Influence of Others on Knowledge & Understanding

Some patients and relatives spoke about seeking advice from their social network regarding their disease and treatment. Sharing insights and experiences with “other people who have had the same illness” was valuable for patients. However, one patient highlighted that his neighbour’s negative experience had led him not to take any medications.

My neighbour was on aspirin. Next thing I know he’s at the hospital with bleeding and then he died. I’ve seen what happened to him and I don’t want to end up like that. (P14, not taking any medications)

Clinicians agreed that social influences played a big role in patients’ lives. Most spoke of the negative media propaganda about statins rather than the influence of the patients’ social networks. However, one HCP felt that negative media influence was only related to specific demographic groups, particularly White British patients, as “they’re the ones reading that information”.

##### Sub-Theme: Language & Health Literacy

All HCPs reported that deprived and non-English speaking patients struggled more with understanding their disease and hence were less adherent.

We found the patients from the lowest economic demographics had a poor understanding of their condition, how and when to seek health, take their meds and so on. (HCP4, vascular surgeon, secondary care)

Additionally, most HCPs highlighted that they found it “challenging to explain the nuances of PAD and management” to non-English speaking patients, even when using an interpreter. A relative of a non-English speaker echoed this, explaining that she had to attend appointments to translate so her mother could understand decisions and participate in her care.

All patients specifically spoke about the need for “layman’s language” to understand their disease. Some patients also suggested how they would prefer their disease explained to them, avoiding medical jargon.

I’m 60 and older people than myself will go in a consultation and they are more confused when they come out than when they went in, because it’s non-words. And they don’t understand anything. And they’ve come, and they don’t know why they’re given a prescription to take, because they didn’t understand what was being told to them in the first place. (P9, high medication adherence)

#### Theme 2: Perceived Treatment Concerns

##### Sub-Theme: Concerns About Medication Side Effects

All patients in this study who were not taking their medications expressed worries about developing side effects, hence decided either not to start, or to stop treatment. Clinicians frequently encountered this concern.

I’ve read the leaflet about the clopidogrel, and now I’m worried it’ll make me bleed inside. What if I end up in the hospital and don’t make it out? I don’t want to take tablets. I don’t think it’s safe. If they can’t fix me without causing side effects, what’s the point? (P14, non-adherent to medications)

Patients are aware of the potential side effects, and they’re worried because they know they’re probably going to get them, so they stop taking their tablets. (HCP12, pharmacist, primary care)

The patients who were non-adherent also expressed concerns about the “long-term impact of medications”, believing that even if they did not have side effects now, they might develop them in the future. Patients who were not on an optimal statin dose reported that they did not wish to be put on a higher, optimal dose, despite being advised to do so by their HCPs, due to their perceived concerns that this would lead to a greater risk of side effects. Clinicians also said that this perception was very frequently reported by their patients.

My cholesterol was quite high. They’ve given me statins. I’ve got to keep taking those and I know I have to take those, but my cholesterol now is ok. So I don’t think I need to get 80 mg, I am quite worried I will get side effects. (P10, high antiplatelet adherence, not-optimised for statins)

I think they’re shocked that they’re going up to such a high dose. And obviously worried about the side effects of that. (HCP14, vascular nurse, secondary care)

##### Sub-Theme: Fear of Damaging Legs

Clinicians noted that many patients feared exercise could worsen claudication or damage their legs, creating a significant barrier to exercise. They emphasised the need to explain that walking through the pain could actually improve symptoms.

The advice to push into pain with claudication is a direct contradiction to the messages around angina in the chest. And yet we would call claudication angina of the leg, in effect, to get some understanding. So it can be scary for patients. (HCP13, podiatrist, primary care)

However, patients did not report having this belief. Only one patient shared this concern, specifically about walking after a stent was inserted.

I am about to have a new stent and I am a bit scared walking after the operation…I just need this stent to work. (P10, high antiplatelet adherence, non-optimised for statins, ex-smoker, does not walk daily)

Most patients reported being concerned about statins, rather than exercise, damaging their legs by “causing more pain and leg cramps”. However, this was not identified by HCPs as a barrier to taking statins.

The problem I have with statins is that they cause trouble with my legs. And I have cramps anyway. I’m wondering if the cramp in my left leg has been worse because of the statins. And I’m wondering if that’s to do with circulatory problems now. I’m not sure. So I don’t want to take them. (P1, not on statins)

#### Theme 3: Motivation

##### Sub-Theme: Fear of Amputation

Most ex-smokers and HCPs reported fear of amputation as a key motivator for quitting smoking. Clinicians also believed it encouraged exercise uptake, however, no patients mentioned this.

I think when if you’re quite blunt with the patients and say, look, you’re on the road to limb loss right now. And I think if you’re honest with them, I think they do get a little bit of a shock and they stop smoking. (HCP12, pharmacist, primary care)

Unfortunately, for some patients, the motivation to quit smoking only materialised after adverse events, specifically an amputation. One amputee reported that losing his leg made him stop smoking, as he was afraid of losing another limb.

##### Sub-Theme: Motivation to Change and Staying on Track

This sub-theme was reported by both patients and HCPs in relation to treatment overall. All patients noted that treatment adherence depended on their motivation to make changes, and stressed the need for a “positive mindset to stay on track”. When they had limited motivation for change, patients could become disengaged with their treatment.

I think the motivation to take on some of the more difficult interventions, such as structured exercise, can sometimes go. And patients drop-out. (HCP15, podiatrist, primary care)

In relation to smoking, all HCPs agreed that patients needed to be motivated to quit. Some patients reported smoking as a coping strategy and a means of stress relief; hence, their motivation to quit was low. However, by staying on track and reminding themselves of the benefits of quitting smoking for their disease, smoking abstinence was often achieved.

Smoking was always my way of coping with life. I relapsed once for about a day where I had about three, because I was quite stressed. I had to stay strong, went cold turkey, and never went back to it. (P9, ex-smoker)

##### Sub-Theme: “An Existence Not a Life”

Most patients talked about the negative impact of PAD on their mental health and motivation. Feeling helpless and stuck contributed to feelings of isolation and low mood. Some patients reported giving up, and one said that living with PAD was “an existence, not a life” (P14). Hence, these patients disengaged from their treatment and continued their unhealthy lifestyles, staying indoors, not wanting to prolong what another described as a miserable life.

All HCPs agreed that PAD significantly impacts patients’ quality of life (QoL). They acknowledged that patients sometimes chose to continue their unhealthy lifestyles, as these might be their only enjoyment in life. They remarked that anxiety and depression often went unsupported due to a lack of psychological services in the NHS. Additionally, HCPs reported that patients often declined to take part in SET due to psychological distress.

I think a lot of people with mental health problems like anxiety, depression, once you want to mention a class, they just shut down because they can’t face going into a room with strangers there. They think, no, no, that’s not for me. (HCP15, podiatrist, primary care)

#### Theme 4: Healthcare Professional Influence

##### Sub-Theme: Trust in Specialist Care

Building rapport and trust between patients and their HCPs was seen as essential for treatment engagement. All patients reported that being treated humanely increased their trust in their HCPs.

I go to my appointments, and, yes, it’s my leg. But I get asked about myself as well. Yes, you’re treating my leg, but you’re asking me, how am I? And that makes you comfortable with the person that’s giving you care. And I can trust them, I know they genuinely care for me. (P9, high medication adherence, ex-smoker, does not walk daily)

Most study participants agreed that patients trusted their secondary care HCPs more than their primary care clinicians. Patients felt that vascular experts knew more about their condition than anyone in primary care, perceiving that the only specialist care available was in secondary care.

There is something that patients value from hearing it from somebody other than their GP. They do value that sort of secondary care opinion. (HCP18, GP, primary care)

##### Sub-Theme: Conflicting Information from HCPs

This sub-theme was mainly reported by patients and HCPs for medications, particularly statins. Conflicting advice from different professionals was seen as confusing, undermining trust, and hindering informed decisions. This was particularly evident in relation to statin optimisation, with vascular clinicians suggesting that statin doses should be increased to the optimal level for secondary CVD prevention, but patients being unwilling to increase the dose. Often, this was because their primary care clinicians had told them they did not need a higher statin dose, since their cholesterol levels were not elevated. Vascular HCPs reported often having to “fight with GPs to increase statin dose”.

But that can be contradicted, that message, sometimes by other health professionals, and including GPs and vascular surgeons, sadly. And we’re constantly having to have debates about that with the clinicians, which doesn’t help patient understanding when they then get contradicting information. (HCP15, primary care)

Even though most patients reported greater trust in their specialist vascular clinician, when they received conflicting information, they often chose to follow the advice of the person offering the more convenient option. This was usually a primary care clinician.

Last time I came to the hospital, there’s a doctor there [vascular] who told me I should take double [statin] dose, but I don’t really listen to the doctor sometimes when they tell me about those tablets. Because I’ve been taking it for I don’t know how many years. My GP said my cholesterol is not high anymore. So, tell me, I should take a double dose now. For what reason? (P2, not optimised for statins)

##### Sub-Theme: Involving Patients in Decision Making

Some HCPs felt that applying a shared decision-making (SDM) approach for the treatment plan could make people “more likely to engage with their treatment”. They also talked about empowering patients to self-manage, however, they stressed that self-management was not appropriate for all patients, especially those with cognitive challenges. None of the interviewed patients or relatives reported wanting SDM. When specifically asked about their feelings regarding involvement in the decision-making process, most felt it was not their place to make decisions, indicating that this should be the doctors’ responsibility. Similarly, HCPs recognised that patients often adopted a *passive approach* when discussing treatment options.

Don’t ask me, ask the doctors. I don’t know. (P2, high antiplatelet adherence, non-optimised for statins, ex-smoker, does not walk daily)

I feel some patients just don’t want to help themselves. The NHS will sort it out. It’s all about shared decision making, but tell me, how we can do this when people don’t care what happens to them?. (HCP5, vascular surgeon, secondary care)

### Practicalities

#### Theme 5: Social & Physical Context

##### Sub-Theme: Social Support

Support from the patients’ social network could either facilitate or hinder treatment adherence. Patients and HCPs often reported that having an engaging and supportive family, or a dog, was an adherence facilitator for overall treatment and exercise.

Having my dog keeps me moving. He needs his walks, and that gets me out every day, even when I don’t feel like it. It’s good for both of us. (P15, walks daily)

I always think it’s good news if a patient’s got a dog because they then have to go out and walk the dog. (HCP18, GP, primary care)

When asked about the type of support patients received from family, the majority reported day-to-day tasks, such as household chores and driving around to medical appointments, whilst one patient reported that his wife was in charge of his medications, which facilitated his medication adherence.

The wife is very supportive and she gives me a good kick up the backside when I get a bit nervous and things like that. She doesn’t crack the whip or anything like that but she’s there to support me and she’s effectively my carer. She does the cleaning and cooking at the house, which is definitely a big help. (P3, high medication adherence, ex-smoker, exercises daily)

I think it definitely helps if we see the patient attending with their partner. I think the attitude of the family and friends around the person definitely makes a difference. If you can get them on board as well, then I think it definitely helps. (HCP1, vascular nurse, secondary care)

##### Sub-Theme: Negative Social Influences

Specifically for smoking, all groups reported that patients who smoked within a social circle found it harder to quit. Some patients who managed to quit also spoke about particular times in their lives, where social or work commitments would have made it impossible to quit smoking.

When I used to work, I would go to the pub and have a fag…Everyone was smoking. So even if I wanted to quit, I think it would have been impossible. (P3, ex-smoker)

I think it’s very hard for one person to quit smoking when the other person living in close proximity is still smoking. (HCP2, vascular surgeon, secondary care)

Some patients also spoke about feelings of social stigma due to their disease, which influenced their decisions about exercise and walking behaviour. They preferred not to leave the house as they did not want to feel “judged” or treated differently by others because of the way they walked.

##### Sub-Theme: Environmental Barriers

Bad weather, prolonged travelling time to exercise facilities and limited time during the day were reported as barriers to exercise by all groups. The motivation to leave the house on a cold and rainy day was limited. Some patients and relatives also reported that it was “safer to stay inside” when it rained.

I think it’s just being motivated to get out when the weather is terrible, they just can’t be bothered. (HCP8, vascular surgeon, secondary care)

Specifically for SET attendance, the logistics around travelling, in terms of time, mode of transport, and parking, were reported as barriers by some relatives and most HCPs.

They referred my mum to the exercise programme, but it’s at [Hospital Name], which is too far. So she can’t go there. It would be nice if there was something closer to us. My mum would be able to go more often. (R4, relative of P12)

So I think some of those things maybe transport, maybe work life balance, maybe childcare, you know, these some of these people are working 2/3 jobs to keep a roof over their head and said being told to go in excess of 30 minutes is difficult to fit in and they just want the seemingly quick fix, an intervention. (HCP4, vascular surgeon, secondary care)

#### Theme 6: Everyday Reality

##### Sub-Theme: Health-Related Challenge

When talking about health-related challenges, all groups spoke about claudication pain and the existence of other comorbidities and how these could affect adherence. Claudication pain was reported as the main barrier to exercise by all patients, with patients being unable to continue walking due to the pain.

The pain in my legs was increasing and I just couldn’t walk as far. The pain is the main thing that stops me. (P10, exercises daily)

I’m sure some people are in so much pain, they do just find it too difficult. (HCP7, vascular scientist, secondary care)

Besides claudication pain, most patients and relatives reported that symptoms related to other conditions, such as arthritis and balance issues, were also barriers to exercise. However, some patients reported doing sitting exercises to prevent falls and to stay active.

When I walk, if there is something on the floor, I don’t notice it, I truly don’t. My ankle bends and I fall. So I avoid walking for long distance. (P6, does not exercise daily)

It’s the arthritis and the back pain, on top of the claudication pain. It’s just hard for her to walk. She doesn’t like taking painkillers, she is trying to use a stick, because she feels safer walking with the stick as a balance to help her walk. (R3, relative of P10)

Most HCPs stressed that patients with PAD have multiple comorbidities; hence, they felt that patients were not always focused on the management of PAD until their symptoms worsened or they had a cardiovascular event. Most HCPs reported that patients continued smoking whilst experiencing CLTI symptoms, and that they were only willing to change their lifestyle and quit smoking after having a heart attack or stroke.

Until they get a heart attack or a stroke, they are unwilling to stop smoking. They don’t care what happens to their legs. They basically are happy to keep smoking with the blue toes. (HCP2, vascular surgeon, secondary care)

##### Sub-Theme: Experiencing Medication Side Effects

Patients and relatives identified medication side effects as a barrier to adherence. Patient participants who had previously experienced side effects reported that they were not willing to continue their medications or try alternative options, as they did not want to continue having side effects.

I used to take statins and I had terrible cramps. So I stopped them and I felt so much better! Then I came to vascular and they put me on statins again. I told the doctor I am not going to take them. (P1, high adherence for antiplatelets, not on statins)

Most HCPs reported that statistically only a small percentage of patients get side effects, and these patients should be offered alternative medications. Hence, some HCPs did not perceive medication side effects as a true adherence barrier, rather an “excuse to not take tablets”.

In my experience, very few patients actually experience side effects. It’s often more about their perception or reluctance, and we can usually offer alternatives if they genuinely have issues. We have so many statin options, ezetimibe, etc. (HCP4, vascular surgeon, secondary care)

##### Sub-Theme: The Power of Habit

Highly adherent patients reported that creating routines helped them stick to medications and exercise. Patients who were taking their medications daily reported strategies, like using a Dossett box, or taking their medications all together, to avoid forgetting them.

Just get up first thing in the morning when I’m having my first cup of tea. My medication is right in front of me, so I just take them. And wherever I get up and sit in the morning, that’s where my medication is. (P9, high medication adherence)

In this study, none of the patients identified forgetfulness as a barrier to medication adherence. Only one patient reported rarely forgetting one of his medications, but he did not feel it was significant enough to need a strategy in place.

All patients who reported walking daily noted that having a walking routine facilitated exercise. Patients described walking at specific times during the day, or after meals, as part of their daily plan.

I try to walk three times a day, first thing in the morning, after lunch and after dinner. I don’t go far, just around the house. It can get boring but it’s something that I do every day and it keeps me going. (P1, walks daily)

On the other hand, patients who smoked or were ex-smokers reported that it was part of their life, and that habits were hard to break.

Human beings are creatures of habit. I’ve been smoking since I was 12-13 years old. It’s part of my life. (P13, recent ex-smoker)

##### Sub-Theme: Getting on with Life

This sub-theme was only reported by patients and their relatives. Despite the huge impact PAD had on patients’ wellbeing and quality of life, some patients reported that they were simply trying to “get on with life” and get used to the new reality. Patients and relatives spoke about “changing things in their lives to avoid the pain”. Some relatives also spoke about patients putting up with the pain and trying to continue their day-to-day life, as they felt there was no other choice. Where this was the case, relatives felt that patients had accepted this new reality of living with PAD and did not wish to seek further medical help.

My husband and I used to take the train to London and go shopping, but after his diagnosis he didn’t feel confident doing that. We started planning our journey differently, allowing more time to get to the station, and taking things slow. He had to accept this new reality and get on with it. (R1, relative of P3)

#### Theme 7: Healthcare System Factors

##### Sub-Theme: Access to Services

This sub-theme was only reported by HCPs to describe the dearth of PAD-specific services, contributing to a lack of patient engagement with their treatment. Most HCPs believed that “patients will engage with what is available”. Many noted that despite guideline recommendations, few UK centres offered SET, and smoking cessation support was also limited, leaving patients without optimal treatment options.

The fact that most of the population doesn’t have access to that [SET] is not acceptable. So we’ve got to think of other ways to work with selling and providing exercise. (HCP15, podiatrist, primary care)

The other challenge that we have with these patients with intermittent claudication is maybe getting them to stop smoking and the access to smoking cessation is not universal. (HCP2, vascular surgeon, secondary care)

Clinicians also reported that even when services were available, long waiting lists were an added barrier to engagement. Interviewed primary care clinicians emphasised that the referral process from primary to secondary care was very lengthy, with patients being “stuck in a black hole”. By the time patients are seen, their symptoms have worsened. All HCPs stressed that these delays also reduced patients’ motivation for lifestyle change.

Unfortunately, when you now refer to the hospital, the patients are not seen. It takes forever, and by the time they get seen, it’s too late. (HCP17, GP, primary care)

All HCPs also spoke about deprived patients and those who did not speak English having more difficulties accessing care. These populations were described as having less insight into their conditions, hence, they were less likely to seek help when necessary.

A huge majority are from a low socioeconomic background. A very high percentage can’t read or write, or one or the other. I just think it’s harder for this population to access services and seek help. (HCP6, physiotherapist, secondary care)

##### Sub-Theme: Ongoing Support & Follow-Up

This sub-theme highlights the challenges faced by both patients and HCPs regarding gaps in continuity of care. Interviewed clinicians working in services where no standard outpatient follow-up was offered for patients with PAD reported that they did not know what happens to patients after they get discharged back to primary care. This meant it was impossible to monitor progress and sustained behaviours. Most HCPs felt that follow-ups would support adherence and continuity, by “reiterating the message”, but admitted that system pressures limited holistic care. Clinicians saw value in virtual monitoring, while patients preferred face-to-face appointments.

All interviewed patients and relatives reported the need for more continuous support, noting that cancelled appointments or no formal follow-up caused frustration and could lead to treatment disengagement.

He told me it might get better, it might get worse, but I was left then with no treatment plan. There was no follow-up or anything at all. It was, there you go, you’ve had your consultation. At the moment I feel that I could do with some one-to-one support. But I haven’t been offered that. (P3, high medication adherence, ex-smoker, exercises daily)

## Discussion

### Main Findings

This is the first study exploring patients’, relatives’ and HCPs’ perceptions of PAD and its management, identifying barriers and facilitators to treatment adherence. The findings highlight treatment concerns, as well as a lack of patient knowledge, motivation, and healthcare system support, all of which negatively affect adherence to medications, exercise, smoking cessation, and long-term disease management. Perceptions between patients/relatives and HCPs did not always align, especially about medication side effects.

### Perceptions

The key theme across all groups was patients’ limited knowledge and understanding of the disease and its treatment. Consistent with previous qualitative research on patients’ perceptions about PAD, some participants considered PAD temporary, and felt that self-management was unnecessary, believing instead that surgery could provide a cure or alleviate symptoms.^33–35^ In our study, limited awareness of PAD as a systemic condition led patients to question the value of long-term pharmacotherapy, particularly statins, often discontinuing treatment when immediate symptom relief was lacking. These findings align with our previous qualitative study in PAD^26^ and studies applying the PaPa framework in stroke and HIV populations.^26^,^36^,^37^

In our study, participants across all groups reported that patients with PAD frequently struggle to quit smoking, even when aware of the risks, which is consistent with prior PAD research.^26^,^38^ Clinicians have previously emphasised that smoking rates typically decline following major cardiovascular or limb events in cardiac patients, due to increased awareness of recurrence risk and mortality,^39^,^40^ which is also in line with our findings. In our study, many patients also viewed exercise as ineffective for improving their condition, echoing findings of previous PAD research.^22^,^41^ A recent umbrella review exploring factors associated with adherence to physical activity in patients with chronic conditions identified that knowledge, expectations, and perceived benefits are key adherence determinants.^42^ These findings highlight the need for clear communication about the systemic risks of PAD, the role of medications and lifestyle changes, and realistic expectations for surgical outcomes.

Communication gaps from HCPs, limited information and low health literacy were central barriers to adherence. Without clear, lay-friendly information, patients sought knowledge from alternative sources, like the internet, social media, and peer networks. Previous research has shown that such media can create uncertainty and confusion around treatment decisions, thereby reducing adherence.^43^,^44^ A meta-analysis identified that effective communication with HCPs strongly improves treatment adherence.^45^

Fear of side effects, especially statins, was a prominent barrier to adherence, reported by patients and their relatives. This finding aligns with other qualitative studies in cardiovascular populations and our previous work on PAD treatment perceptions.^26^,^36^,^44^,^46^ In line with our findings, another qualitative study identified fear of pain when walking as a key exercise barrier.^34^ This highlights knowledge and communication gaps around the safety and therapeutic value of exercise.

Adherence to PAD treatment is influenced by motivational factors. Consistent with previous research, this study found that self-motivation, establishing routines, and a positive mindset facilitated engagement, while unreadiness to change and negative treatment attitudes acted as barriers.^22^,^26^,^47^ Fear of amputation emerged as a strong motivator for smoking cessation, supporting previous findings that perceived threat can drive behaviour change.^26^,^39^,^40^ Moreover, PAD imposes a significant psychological burden, with many patients describing that they did not feel they were fully living life. Other studies have found that anxiety, depression, and social isolation reduced motivation for lifestyle changes and supervised exercise therapy.^48–50^ These findings underscore the importance of integrating psychological support into chronic disease management to enhance adherence.

Undoubtedly, HCPs play a significant role in patient adherence. Trust in specialist vascular clinicians facilitated adherence, while conflicting advice from different professionals reduced adherence, particularly regarding statin optimisation. Previous research has shown that trust in specialist care is associated with greater adherence to chronic disease management.^51^ However, interprofessional disagreement between primary and secondary care remains a barrier in chronic disease management.^52^

### Practicalities

Our study showed that environmental and social factors can also influence adherence. These findings are in line with systematic reviews^22^,^41^ and our previous qualitative study in PAD,^26^ suggesting that practical barriers such as cold weather, rain, and limited access to exercise facilities often lead patients to discontinue activity. Existing research is consistent with our findings that having social support does not always lead to better health behaviours, highlighting the complex nature of social influence.^53^ The presence of companionship and family support enables medication use, physical activity, and smoking cessation;^26^,^44^,^54^,^55^ while negative behaviours, such as nagging or not offering support, may undermine efforts.^56^ Similarly, in our study, experiences and advice from others strongly affected statin adherence. Specifically, hearing about side effects raised anxieties, leading to medication non-initiation or discontinuation.^44^

Participants often spoke about the everyday reality of living with PAD and how this could influence adherence. In line with our findings, other studies have reported that claudication pain and other comorbidities limited physical activity.^22^,^26^,^41^ While polypharmacy is often cited as a barrier in chronic disease,^44^ patients in our study did not report this. Although HCPs in our study were sceptical that patients were actually experiencing side effects, literature on chronic disease shows strong links between side effects and non-adherence.^26^,^44^ Similar to our study, a qualitative focus group study of 94 HCPs looking after patients with polypharmacy found that clinicians reported patients often stop medications they believe cause side effects.^57^ Clinicians perceived that this was due to patients’ medication misconceptions, rather than actual side effects.^57^

Our findings are in line with previous literature suggesting that insufficient healthcare provision is an adherence barrier for statins.^44^ Currently, there are persistent gaps in UK PAD service provision, with care remaining inconsistent despite guideline recommendations.^58^ Implementing structured follow-up could reinforce education, support habit formation, and improve patient engagement in long-term disease management.

To summarise, the seven themes clustered into perceptual and practical determinants of adherence, clarifying both mechanisms and intervention targets. Knowledge, treatment concerns, motivation, and HCP influence reflected perceptual barriers shaping beliefs about PAD, treatment necessity, and risks, indicating a need for targeted educational interventions. In contrast, social and physical contexts, everyday realities, and healthcare system factors posed practical barriers that limited patients’ ability to act on their intentions, highlighting the importance of structured support, coordinated care, and accessible services. Several themes spanned both domains, demonstrating that adherence is shaped by interacting influences rather than single factors. This mapping underscores the need for multi-component interventions that simultaneously address perceptions, capabilities, and contextual barriers to optimise long-term PAD management.

## Limitations

This study has some limitations. First, self-selection bias may have occurred, as patients and relatives who chose to participate may have been more engaged than the wider PAD population. Similarly, HCPs with a specialist interest in PAD care may have been more inclined to take part. The findings are also specific to the NHS England context and may not be generalisable to other healthcare systems. Finally, only a small proportion of patients (4/15) had experienced a previous chronic critical limb ischaemia; the results may not reflect the experiences of patients with more severe PAD.

## Conclusion

This is the first qualitative study to explore treatment adherence in PAD from the perspectives of patients, relatives, and HCPs. Our findings reveal that adherence is shaped by multifaceted influences, including patient knowledge, motivation, social and environmental influences, and healthcare system structures. Misconceptions of PAD as an acute rather than chronic, systemic condition, concerns about medication side effects, claudication pain, and inconsistent professional advice undermined adherence, while trust in specialist care, daily routines, and supportive relationships facilitated it. Limited communication, information resources, health literacy, and service provision further hindered long-term management. Addressing these challenges requires a patient-centred approach, combining clear education, psychological and social support, consistent interprofessional communication, and structured follow-up. Embedding these strategies into care pathways could improve adherence, clinical outcomes, and quality of life for patients with PAD.

## Data Availability

The data that support the findings of this study are available on request from the corresponding author. The data are not publicly available due to privacy or ethical restrictions.
